# Western Medical Convention

**Published:** 1848

**Authors:** 


### ARTICLE V.

### WESTERN MEDICAL CONVENTION.

The Western Lancet for January last, contains an article suggesting the propriety of a convention of delegates from Western Medical Colleges, to consult upon certain points of interest particularly to them, and to consider the propriety of adopting and carrying out the suggestions of the National medical convention.

The proposition, so far as we could learn, met with favor generally, and would likely have been carried into effect, but for the shortness of time between the notice and the time proposed for meeting. We, however, are not informed how far the call was responded to by the selection of delegates, except that the faculty of Rush Medical College made such appointment; but we see many reasons why such a convention should be held, and no good reason against it.

West of New York, and North of Tennessee, there are ten medical schools, viz.: one at Lexington, and one at Louisville, Kentucky; one at Cincinnati, one at Columbus, and one at Cleveland, Ohio; one at Laporte, Indiana; one at Chicago, and one at Jacksonville, Illinois; and two at St. Louis, Missouri; amongst which there is great want of uniformity in organization, requirements, and policy.

There may be points of difference which the customs of the schools, and the expectations of the profession in the different latitudes of this district of country, may require to be retained. But if by meeting in convention these matters could be so harmonized as to bring about uniformity in other respects, it would be a most desirable end attained. E.

